# Post-vaccination SARS-CoV-2 seroprevalence in children aged 3-11 years and the positivity in unvaccinated children: A retrospective, single-center study

**DOI:** 10.3389/fimmu.2022.1030238

**Published:** 2022-11-07

**Authors:** Jing Li, Menglei Ge, Shuzhi Dai, Qinwei Song, Weijie Liu, Ying Wang, Wenjian Xu, Lijuan Ma

**Affiliations:** Department of Clinical Laboratory, Children’s Hospital Affiliated to Capital Institute of Pediatrics, Beijing, China

**Keywords:** children, COVID-19, vaccination, antibody, SARS-CoV-2

## Abstract

**Objective:**

To analyze the positivity and levels of SARS-CoV-2 antibodies in vaccinated children to evaluate the humoral immune response of vaccination on pediatric population. Analysis on the causes of antibody positivity in unvaccinated children.

**Methods:**

A retrospective study was conducted on children who were admitted to the Children’s Hospital Affiliated to Capital Institute of Pediatrics. The clinical data of serological testing of SARS-CoV-2 immunoglobulin M (IgM) and IgG antibodies were collected from SARS-CoV-2 vaccinated or unvaccinated children with no evidence of prior novel coronavirus infection. Chemiluminescence immunoassay was utilized for the *in vitro* determination of SARS-CoV-2 antibodies.

**Results:**

A total of 3,321 healthy children aged 6-11 years received two doses of inactivated SARS-CoV-2 vaccine. At 1 month after the second dose, the positive rate (96.5%) and levels [8.039 (interquartile range (IQR), 6.067-9.098)] of SARS-CoV-2 IgG antibodies reached the peak and remained at a high level for 2-3 months, after which the positive rate and level of vaccine-induced IgG antibody gradually decreased. Compared with 1 month after the second dose of vaccine, the positive rate of IgG antibody decreased to 70.4% at 7 months, and the antibody level decreased by 69.0%. A total of 945 children aged 3-5 years received one or two doses of inactivated vaccine. The positive rate and levels of SARS-CoV-2 IgG antibody in participants remained high for 3 months after vaccination. There was no gender-based difference in positive rate of IgG antibody in children aged 3-11 years old (P>0.05). Among the 5,309 unvaccinated children aged 0 day to 11 years, 105 (2.0%) were positive for SARS-CoV-2 IgG antibody, which was associated with passive infusion. The maternal humoral response to COVID-19 vaccination in noninfected pregnant women was transferred through the placenta to the fetus, and some children obtained SARS-CoV-2-positive antibodies through blood transfusion.

**Conclusions:**

Inactivated SARS-CoV-2 vaccines could induce robust humoral immune response that gradually declined within several months after the second dose. Therefore, it helps to determine whether children receive a booster dose and elicit a long-term memory immune response. Positive SARS-CoV-2 antibodies in unvaccinated children were associated with passive IgG antibody infusion.

## Introduction

Since December 2019, coronavirus disease 2019 (COVID-19), caused by the novel severe acute respiratory syndrome coronavirus 2 (SARS-CoV-2), has spread across the globe and emerged as the pandemic even among children (1, 2). Initial epidemiological data indicated the majority of children had milder symptoms of COVID-19 infection than adults. A study revealed 171 children with confirmed disease presented more detailed symptoms. The most common symptoms were cough (48.5%) and fever of at least 37.5°C (41.5%) (3). Moreover, another meta‐analysis found that cough and fever occurred in up to 63.4% and 92.8% of adults, respectively (4). Recent epidemiologic data seem to suggest that children with SARS-CoV-2 infection were at greater risk for hospitalization or admission to a pediatric intensive care unit (PICU) during the acute phase of illness than the initial evaluation. Notably, hospitalizations and deaths were partly due to the multisystem inflammatory syndrome (MIS-C) associated with SARS-CoV-2, which led to serious and life-threatening illness in previously healthy children and adolescents (5, 6).

SARS-CoV-2 enters the host cell by binding to host receptors *via* S protein. The S1 domain of spike protein acts as a major surface antigen. It contains two subunits, including N‐terminal domain (NTD) and C‐terminal domain (CTD). The S1‐CTD acts as a receptor‐binding domain (RBD). The RBD interacts with the 18 residues of angiotensin-converting enzyme-2 (ACE‐2). The S2 domain is a membrane fusion subunit, which mediates the fusion of virus and host cell membrane through conformational changes, allowing viral RNAs to enter cells and replicate (7, 8). The RBD of S1 subunit is the target of most neutralizing antibody (9, 10). A potent neutralizing antibody binds to RBD and blocks its interaction with host cell ACE2 receptor.

Vaccines for COVID-19 have been developed and approved quickly, which assisted governments to better control the spread of SARS-CoV-2. An mRNA vaccine candidate BNT162b2 (Tozinameran; Pfizer–BioNTech) has shown 100% efficacy in a population aged 12–25 years (11). Another vaccine candidate mRNA-1273 (Moderna, Cambridge, MA, USA) is reported to elicit serological response in at least 99.0% of the participants aged 6-11 years one month after the second dose (12). The immunogenicity of the inactivated vaccine CoronaVac (Sinovac, Beijing, China) and BBIBP-CorV (Beijing Institute of Biological Products, Beijing, China) have been tested in a phase 1/2 trial in a population aged 3–17 years. Both inactivated vaccines were immunogenic and induced robust humoral responses, with a seroconversion ratio of 100% in all vaccination individuals at 28 days after the second dose (13, 14). From November 2021, BBIBP-CorV (4μg, 0.5ml per dose) and CoronaVac (600SU, 0.5ml per dose) were approved for emergency use for children older than 3 years in China. A complete vaccine schedule was given in two doses, 28 days apart, intramuscularly in the deltoid muscle.

However, in the real-world evaluation, few studies have concentrated on the humoral immunity response of inactivated SARS-CoV-2 vaccines on healthy children aged under 11 years for a moderate duration. In the present study, data of serum SARS-CoV-2 antibodies from healthy children admitted to the Children’s Hospital, Capital Institute of Pediatrics (Beijing, China) were retrospectively analyzed. This study aimed to evaluate the positivity and levels of SARS-CoV-2 serological antibodies in vaccinated pediatric population, and analyze the causes of positive antibodies detected in unvaccinated population, so as to facilitate accurate clinical diagnosis.

## Methods

### Study design and participants

**Figure 1:**
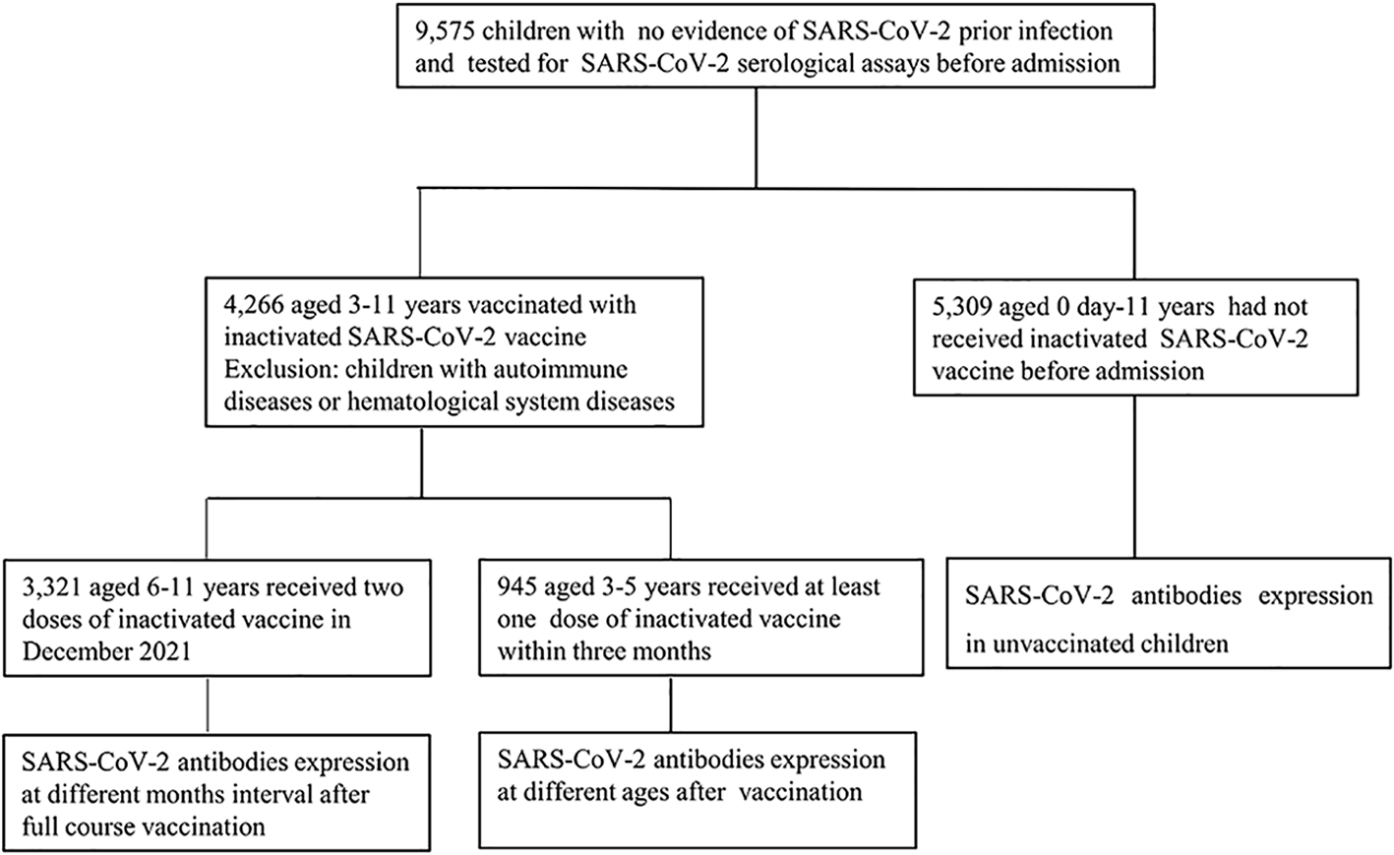
Study involvement and group division (January 2022 -July 2022). Children admitted to the Children’s Hospital, Capital Institute of Pediatrics with no evidence of SARS-CoV-2 prior infection were enrolled in the study. All the children tested for SARS-CoV-2 serological assay. In the vaccinated group, children with autoimmune diseases or hematological system diseases were excluded.

Data of SARS-CoV-2 immunoglobulin M (IgM) and IgG serological assays collected from children who were admitted to the Children’s Hospital, Capital Institute of Pediatrics (a 400-bed tertiary pediatric hospital in China with an average of 30,000 admissions per year) from January 2022 to July 2022 were retrospectively analyzed. All the participants had no history of COVID-19 prior exposure. Due to the epidemic prevention and control measures, all children who need to be hospitalized should be tested for 2019-nCoV nucleic acid and SARS-CoV-2 serological assays before admission within 72 hours. Based on whether children had received the inactivated SARS-CoV-2 vaccines, they were divided into vaccinated group (3-11 years) and unvaccinated group (0 day-11 years). In the vaccinated group, Children were divided into 6-11 years group who had received two doses vaccine and 3-5 years group who had received at least one dose vaccine. Considering the hematological products transfusion may affect seropositive rates in the vaccinated individuals, children with autoimmune diseases or hematological system diseases were excluded (**Figure 1**).This was a retrospective study, and all the participants’ data were anonymously reported. The study protocol was approved by the Ethics Committee of Capital Institute of Pediatrics (approval number SHERLLM2022029).

### Laboratory assays

Approximately 3 ml blood was collected in coagulation tubes from all individuals by venipuncture. Serum was separated after centrifugation at 3,000 rpm/min for 10 min, and it was analyzed for the SARS-CoV-2 IgM and IgG antibodies using the reagent matching the Maccura i1000 automatic chemiluminescence analyzer (Maccura Biotechnology Co., Ltd., Chengdu, China). Magnetic microparticle chemiluminescence immunoassay was used for the *in vitro* determination of IgG/IgM antibodies to the recombinant RBD antigen of SARS-CoV-2 Spike protein. Detection procedures were performed according to manufacturer’s instructions. The resulting chemiluminescent reaction was measured as relative luminescence units (RLUs). It was proportional to the content of SARS-CoV-2 IgG/IgM antibodies in the specimen. The SARS-CoV-2 IgG assay was based on the indirect method. Specific IgG antibodies in the sample bind to magnetic microparticles coated with SARS-CoV-2 recombinant antigens. Then, the immune conjugate is combined with the mouse anti-human IgG antibodies labelled with acridinium ester. Specific IgM antibodies in the specimen bind to magnetic microparticles coated with mouse anti-human IgM monoclonal antibodies to form an immune conjugate, which is combined with the SARS-CoV-2 recombinant antigens labelled with acridinium ester. The serological assay is semiquantitative and the concentration was recorded as S/CO(RLU of samples to be tested/cutoff), S/CO<1.000 indicated a negative result, and S/CO≥1.000 represented a positive result.

### Statistical Analysis

Continuous variables were presented as median and interquartile range (IQR). Independent continuous variables were compared using the Mann‐Whitney U test. Categorical variables were expressed as number and percentage, and compared using the χ2 test. P < 0.05 was considered statistically significant. The statistical analysis and illustration of graphs were performed by GraphPad Prism 8.0 software (GraphPad Software Inc., San Diego, CA, USA).

## Results

### Children’s characteristics

The data of SARS-CoV-2 IgM and IgG antibodies from 9,575 children who were admitted to the Children’s Hospital, Capital Institute of Pediatrics from January 2022 to July 2022 were retrospectively analyzed. A total of 4,266 children were vaccinated with inactivated SARS-CoV-2 vaccines, including 3,321 children aged 6-11 years and received two doses of vaccine in December 2021, as well as 945 children aged 3-5 years received at least one dose of inactivated SARS-CoV-2 vaccine. Besides, 5,309 children aged 0-11 years old had not received SARS-CoV-2 vaccines. In the vaccinated group, there were 2,571 males and 1,695 females; 3,012 males and 2,297 females were involved in the unvaccinated group (**Figure 1**, **Table 1**).

**Table 1:** Gender and age distributions of children who were tested for SARS-CoV-2 IgM and IgG antibodies.

Group	Age	Post vaccination (months or age)	Number of cases	Gender	
				Male	Female
Vaccinated with 2 doses	6-11 years (N=3,321)	After 1 month	689	405	284
		After 2 months	535	349	186
		After 3 months	414	227	187
		After 4 months	395	218	177
		After 5 months	354	196	158
		After 6 months	434	276	158
		After 7 months	500	330	170
Vaccinated with at least 1 dose	3-5 years (N=945)	3 years	224	141	83
		4 years	347	210	137
		5 years	374	219	155
Unvaccinated	0 day-11 years (N=5,309)	0-28 days	473	284	189
		1 month-2 years	2,504	1,361	1,143
		3-5 years	1,524	875	649
		6-11 years	808	492	316
Total			9,575	5,583	3,992

Data were presented as number. Vaccinated with 2 doses group: children aged 6-11 years old were vaccinated with 2 doses of inactivated SARS-CoV-2 vaccines. According to the interval between the detection of SARS-CoV-2 IgM and IgG antibodies and the second dose of vaccines, it was divided into 7 periods from 1 to 7 months after vaccination.

Vaccinated with at least 1 dose group: children aged 3-5 years old were vaccinated with at least 1 dose of inactivated SARS-CoV-2 vaccines. According to the age, the group was divided into three age-based subgroups. Unvaccinated group: pediatric population who did not receive inactivated SARS-CoV-2 vaccines.

### The levels of antibodies at different months after vaccinated with two doses in participants aged 6-11 years

**Figure 2:**
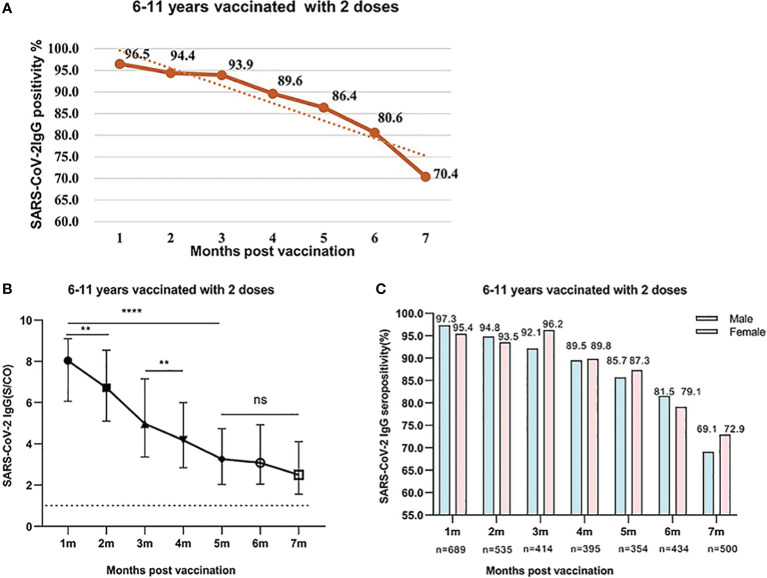
**(A)** SARS-CoV-2 IgG seropositivity in participants who were aged 6-11 years old and were vaccinated with two doses of vaccines after 1-7 months. Data were presented as percentage. **(B)** SARS-CoV-2 IgG levels in participants who were aged 6-11 years and were vaccinated with two doses of inactivated SARS-CoV-2 vaccines after 1-7 months. Data were expressed as median (interquartile range) and analyzed using the Mann‐Whitney U test. The positive levels of SARS-CoV-2 IgG antibody showed a significant difference among different periods after vaccination (P<0.001). The signs represent median values, and the bands represent interquartile ranges [1 month, 8.039 (6.067-9.098); 2 months, 6.721 (5.094-8.540); 3 months, 4.968 (3.368-7.156); 4 months, 4.183 (2.838-5.998); 5 months, 3.259 (2.03-4.731); 6 months, 3.08 (2.048-4.924); 7 months, 2.492 (1.557-4.116). ns, not significant, **P<0.01, ****P<0.0001]. **(C)** Comparison of SARS-CoV-2 IgG seropositivity between male and female participants who were aged 6-11 years old and were vaccinated with two doses of vaccines. Data were expressed as percentage and analyzed using the Chi-square test. The SARS-CoV-2 IgG seropositivity showed no significant difference between male and female participants. (1 month, χ2 = 2.229, P=0.126; 2 months, χ2 = 0.226, P=0.635; 3 months, χ2 = 2.323, P=0.127; 4 months, χ2 = 0.003, P=0.953; 5 months, χ2 = 0.198, P=0.657; 6 months, χ2 = 0.373, P=0.541; 7 months, χ2 = 0.798, P=0.372).

We analyzed the levels of SARS-CoV-2 IgG and IgM antibodies within 7 months after the second dose. A total of 3,321 healthy children aged 6-11 years were fully vaccinated with inactivated SARS-CoV-2 vaccine in December 2021. According to the interval between the detection time of SARS-CoV-2 IgM and IgG antibodies after the second dose, it was divided into 7 periods from 1 to 7 months. The positive rate of IgG antibody reached a peak at 96.5% after 1 month and maintained high for 3 months, which was above 93.9%.Then, it began to decrease to 89.6%,86.4% at 4 and 5 months respectively. At 6 and 7 months, the positive rate of IgG antibody declined to 80.6% and 70.4%, respectively. From the data above, it can be seen that the seropositivity of IgG antibody gradually decreased over time after the second dose of inactivated SARS-CoV-2 vaccine, and there were significant differences in different periods (χ^2^ = 251.8, P<0.001) (**Figure 2A**).

The response and dynamic levels of IgG antibody were similar to the seropositivity rate. The concentration of IgG reached the peak at 1 month and remained high levels within 2 months. The levels of IgG from 1 to 2 months were 8.039 (IQR, 6.067-9.098), 6.721 (IQR, 5.094-8.540), respectively. With the prolongation of vaccination time, the levels of IgG gradually decreased and remained at moderate level at 3 and 4 months, which were 4.968 (IQR 3.368-7.156) and 4.183 (IQR, 2.838-5.998), respectively. However, the IgG antibody levels at 5-7 months further reduced to 3.259 (IQR, 2.03-4.731), 3.08 (IQR, 2.048-4.924), and 2.492 (1.557-4.116), respectively. After the second dose vaccination, the IgG antibody levels at 5-7 months were significantly lower than those after 1-2 months (All P<0.001) (**Figure 2B**). At the same period after vaccination, the positive rate of IgG antibody in boys and girls was similar, and there was no significant difference between male and female participants (All P>0.05) (**Figure 2C**).

**Figure 3:**
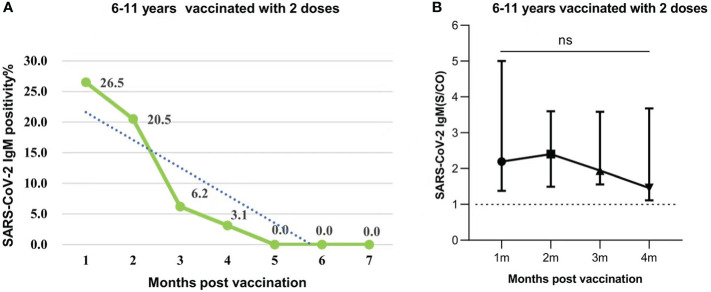
**(A)** SARS-CoV-2 IgM seropositivity in participants who were aged 6-11 years old and were vaccinated with 2 doses of vaccines after 1-7 months. Data were presented as percentage. **(B)** SARS-CoV-2 IgM antibody levels in participants aged 6-11 years injected with 2 doses post vaccination 1-4 months. Data were expressed as median (interquartile range), and analyzed using the Mann‐Whitney U test. The positive levels of SARS-CoV-2 IgM antibody showed no significant difference among different periods (months)(P=0.2051). The signs represent median values, and the lines represent interquartile ranges [1 month, 2.194 (IQR, 1.379-5.003); 2 months, 2.401 (IQR, 1.493-3.599); 3 months, 1.944 (IQR, 1.556-3.582); 4 months, 1.457 (IQR, 1.114-3.678)]. ns, not significant.

The response and duration of SARS-CoV-2 IgM antibody was much different from those of IgG antibody. The positive rate of SARS-CoV-2 IgM antibody was 26.5%, 20.5% at 1 and 2 months, which then dropped to a minimum of 6.2%, 3.1% at 3 and 4 months respectively after the second dose vaccination. None IgM antibody was detected from 5 to 7 months (**Figure 3A**). Further analysis indicated that there was no significant difference in the IgM levels from 1 to 4 months after the second dose (P=0.2051) (**Figure 3B**).

### The levels of antibodies at different ages after received one or two doses of vaccines in participants aged 3-5 years

**Figure 4:**
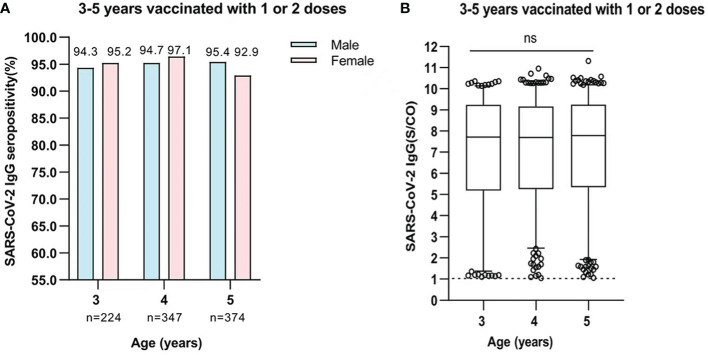
**(A)** Comparison of SARS-CoV-2 IgG seropositivity between male and female participants who were aged 3-5 years and were vaccinated with one or two doses. Data was expressed as percentage, and analyzed using the Chi-square test. The SARS-CoV-2 IgG seropositivity in each age showed no significant difference between male and female participants. (3 years, χ2 = 0.075, P=0.784; 4 years, χ2 = 1.52, P=0.263; 5 years, χ2 = 1.097, P=0.295). **(B)** The levels of SARS-CoV-2 IgG antibody in participants who were aged 3-5 years and were vaccinated with one or two doses. Data was expressed as median (interquartile range), and analyzed using the Mann‐Whitney U test. The midlines represent median values, and the boxes represent interquartile ranges [3 years, 7.712 (IQR, 5.189-9.245); 4 years, 7.694 (IQR, 2.252-9.163); 5 years, 7.780 (IQR, 5.350-9.255)]. The positive levels of SARS-CoV-2 IgG antibody showed no significant difference among participants(P=0.4717). ns, not significant.

A total of 945 participants who were aged 3-5 years old were vaccinated with one or two doses of inactivated SARS-CoV-2 vaccines. Within 3 months after vaccination, the positive rate of SARS-CoV-2 IgG antibody was 94.6% (212/224), 95.6% (332/347), and 94.4% (353/374) in participants who were aged 3, 4, and 5 years, respectively. The seropositivity rate of IgG antibodies produced after vaccination were similar between boys and girls, and there was no significant gender-based difference (**Figure 4A**). The levels of SARS-CoV-2 IgG antibody in participants remained high for 3 months after vaccination. The medium levels of IgG antibody in participants aged 3-5 years were 7.712 (IQR, 5.189-9.245), 7.694 (IQR, 5.292-9.163), and 7.780 (IQR, 5.350-9.255), respectively. There was no significant difference in levels of IgG antibody among different age-based groups (All P>0.05) (**Figure 4B**).

### Positive levels of SARS-CoV-2 IgG antibodies in unvaccinated children

**Figure 5:**
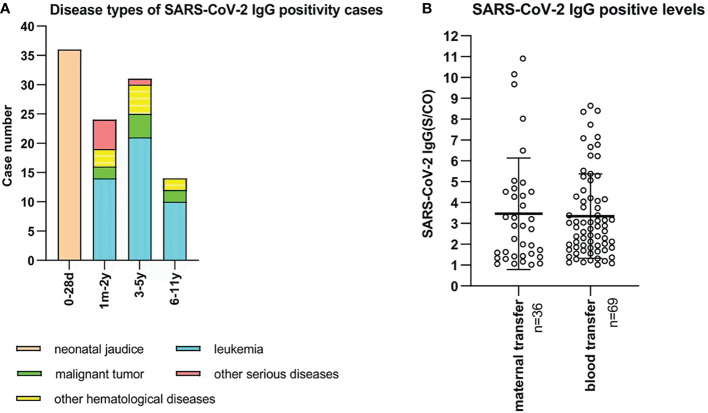
**(A)** Disease types of 105 SARS-CoV-2 IgG positive unvaccinated cases. X-bar represents age, and Y-bar represents the number of cases. 36 neonates were diagnosed with neonatal jaundice. 45, 10, 8, and 6 cases were diagnosed with leukemia, other hematological diseases, malignant tumors, and other serious diseases, respectively. **(B)** SARS-CoV-2 IgG-positive levels of 105 unvaccinated children. Maternal IgG antibody was transferred in 36 cases who were aged 0 to 28 days. Blood products were infused into 69 cases who were aged 1 month to 11 years old.

In the present study, we collected data of 5,309 SARS-CoV-2 antibody tests from unvaccinated children aged from 0 day to 11 years. Serological tests demonstrated 105 positive cases (2.0%, 105/5,309), and all were positive for IgG antibodies. Among 473 neonates, 36 (7.6%) cases were positive for SARS-CoV-2 IgG antibody with neonatal jaundice. The mean age of the neonates was 6.5 (range, 2-28) days, and the IgG antibody levels(S/CO) were 1.064-10.909. These neonates’ mothers received two doses or a booster dose of the inactivated SARS-CoV-2 vaccine before pregnancy or in early pregnancy, and maternal IgG antibody was transferred to the fetus through the placenta. In addition, 69 cases with positive IgG antibody were diagnosed with leukemia, hematological diseases, malignant tumors, or severe systemic diseases. The 69 children included 24 (0.9%, 24/2504) aged 1 month to 2 years, 31 (2.0%, 31/1524) aged 3 to 5 years, and 14 (1.7%, 14/808) aged 6 to 11 years. These children were mainly treated with chemotherapy for hematological diseases or tumors, which resulted in bone marrow suppression. Blood products should be infused to replenish red blood cells, platelets or plasma. SARS-CoV-2 antibodies were detectable in the blood of vaccinated blood donors. Children obtained SARS-CoV-2 antibodies from donors passively through blood transfusion. (**Figures 5A, B**).

### Discussion

Compared with adults, children infected with SARS-CoV-2 generally present with milder symptoms. However, they are still susceptible to SARS-CoV-2 and can also transmit the virus. Therefore, vaccination of all age groups is necessary to curtail the pandemic. Although a large number of vaccines have been shown to elicit robust immune responses, it remains critical for host protection against COVID-19 in adults and adolescents (15, 16). A phase 3 trial of the inactivated COVID-19 vaccine candidates has demonstrated their efficacies in a general population of adults. In Turkey the CoronaVac showed 89.7% vaccine recipients aged 18-59 years were seropositive for RBD-specific total antibody 14 days after the second dose. As well, virus neutralization assays in selected samples from seropositive participants showed a seroconversion rate of at least 92.0% (17). A 6-month study on efficacy of the BNT162b2 mRNA Covid-19 vaccine in participants aged 12 years or older showed efficacy peaked at 96.2% during the interval from 7 days to less than 2 months after the second dose, and declined gradually to 83.7% from 4 months-an average decline of approximately 6% every 2 months (18). However, the data for evaluation of a moderate duration of the humoral immune response in young children are limited. An inactivated vaccine uses the whole virus, essentially retaining the immunogenicity, while lacking infectivity. Vaccination with an inactivated virus can stimulate the body to generate a humoral immune response to defend against SARS-CoV-2 (19). In the present study, SARS-CoV-2 antibodies from healthy children aged 6-11 years who received two doses of inactivated SARS-CoV-2 vaccines were retrospectively analyzed. It was found that 1 month after the second dose vaccine, the positive rate of SARS-CoV-2 IgG antibody reached a peak at 95.6% and remained high for 3 months. From 4 months it declined to 89.6% and then gradually decreased with the prolongation. The positive rate of IgG antibody significantly decreased to 70.4% at 7 months. The dynamic levels of IgG antibody were similar to the seropositivity rate after the second dose. There was a gradual decline in the levels of IgG antibody throughout 7 months. Compared with 1 month after the second dose of vaccine, IgG antibody levels decreased by 26%, 53.2% and 69.0% at 3, 5 and 7 months, respectively. These results revealed that vaccination could induce strong humoral immune responses. In addition, a gradual decline in vaccine induced IgG positive rate and levels was observed. It helps to determine whether a booster is likely to be beneficial after a longer interval and stimulates long-term immune memory response. Among the participants aged 3-5 years within 3 months after at least one dose vaccination, the majority of children developed robust humoral immune response, and the high positive rate and levels of IgG antibodies were recorded. Between male and female participants, the present study showed that the positive rate of IgG antibody was similar after vaccination, suggesting that vaccination was equally effective in eliciting an immune response in boys and girls. A multicenter study recruited 360 healthy adults aged 20-74 years, and they received the first, second, and booster doses of inactivated Sinopharm/BBIBP COVID‐19 vaccines at 0, 1, and 7 months, respectively. The titers of SARS‐CoV‐2 IgG and neutralizing antibodies increased to high levels at the first month after receiving the second dose and declined slowly thereafter. The significant peak of the antibody titer levels after injection of the booster dose highlights the recall immune response (20). A study on children aged 3-12 years received two doses of inactivated SARS-CoV-2 vaccines in Bahrain showed that vaccination led to a promising immunogenicity. It induced an immune response and higher neutralizing antibody titers in children at 5 weeks after vaccination. Meanwhile, vaccination was found equally effective in eliciting an immune response in boys and girls (21). In addition, another study on the efficacy of vaccination in adults showed that among vaccinated individuals with prior evidence of infection, there were no significant differences in IgG levels between males and females across all age groups (22). Another study on seroprevalence of IgG antibody against SARS-CoV-2 conducted in Cyprus showed that, without evidence of prior infection, anti-spike IgG antibodies were detected after injection of 2 doses of mRNA vaccines in 91.2% participants, while antibody levels showed a decrease at 120 days after vaccination (23).

Serological tests can provide information on immune status after vaccination. While the virus neutralizing test is used to directly determine immune function, it is not suitable as a routine test in clinical laboratories due to its complexity and the risks associated with live viruses. We detected IgG antibodies specific to the receptor-binding domain (RBD) of the spike protein on SARS-CoV-2 to evaluate immune response post vaccination. AntiS1-SARS-CoV-2 IgG antibodies showed a correlation with neutralizing antibodies and can be used to estimate the presence of protective immunity in asymptomatic and previously symptomatic SARS-CoV-2 infection (24, 25).

As to individuals with SARS-CoV-2 infection, a specific IgM is the early antibody response that starts and reaches the peak within 7 days after infection. IgM maintenances as long as the acute phase of the disease continues (26). It has been shown that the IgM antibody level was positively correlated with viral load of SARS‐CoV‐2. In addition, a significant reduction of IgM antibody levels was reported as SARS‐CoV‐2 nucleic acid test would be negative (27). In the present study, we analyzed the response of IgM antibodies in 6-11 years healthy children after two doses of inactivated vaccine. A short duration of IgM was observed, which was then decayed within 4 months. The result was consistent with another study which evaluated the response and duration of IgM antibodies in 61 adult participants after inactivated vaccination within 160 days (28).

We retrospectively analyzed the serological data of 5,309 children who had not received inactivated SARS-CoV-2 vaccines, of whom 2.0% were positive for IgG antibodies. Passive transfusion of blood or blood products is the cause of seropositivity for IgG antibodies. A study showed that maternal humoral response to SARS-CoV-2 vaccines in noninfected pregnant women was transferred through the placenta to the fetus, leading to potentially protective immunity for the newborn (29). Meanwhile, the serological response in the cord blood was positively correlated with the maternal humoral response to SARS-CoV-2 IgG antibody. Studies showed that maternal vaccination with two doses of mRNA vaccines was associated with a reduced risk of hospitalization for COVID-19, including critical illness, among infants who were younger than 6 months (30, 31).

In the early phase of the COVID-19 epidemic, transfusion of convalescent plasma from acute coronavirus infection was reported as an effective treatment for severe patients (32). Some studies demonstrated that the response of antibodies to the RBD of the spike protein was highly correlated with neutralization of SARS-CoV-2 (33, 34). As the blood donor vaccination leads to the presence of detectable SARS-CoV-2 antibodies in their blood products. Children obtained SARS-CoV-2 antibodies from donors passively through blood transfusion.

Our report has some limitations. First, this is a retrospective, single-center study to investigate the SARS-CoV-2 antibody responses of vaccinated and unvaccinated children. Second, semiquantitative immunoassay was used to detect SARS-CoV-2 IgG antibodies and the effectiveness of vaccination was not assessed by detecting neutralizing antibodies. However, the same chemiluminescent method was used to monitor antibody production of 22 volunteers at the end of 1, 3 and 6 months after two doses of inactivated vaccine and IgG levels were significantly correlated with neutralizing antibodies, with a correlation coefficient of 0.862 (35). Third, the data on maternal vaccination status were absent to assess the association between neonatal SARS-CoV-2 IgG antibody levels and maternal vaccination.

### Conclusion

Determination of SARS-CoV-2 IgG antibody levels is essential for evaluating vaccine development and strategies in pediatric population. In this real-world, large-scale pediatric population study, it was shown that two doses of inactivated SARS-CoV-2 vaccine in healthy children aged 6-11 years induced a strong humoral immune response that gradually declined within 7 months after the second dose. The high positive rate and levels of IgG antibodies were maintained for 2-3 months, after which a gradual decrease in the positive rate and level of vaccine-induced IgG antibodies was observed. The positive rate of IgG antibody at 7 months after inoculation was only 70%, and the antibody level decreased by 69.0% compared with that at 1 month after the second dose. Therefore, it helps to determine whether children receive a booster dose of vaccine and elicit a long-term memory immune response. Positive SARS-CoV-2 antibodies in unvaccinated children were associated with passive IgG antibody infusion. In conclusion, our study provides evidence of humoral immunity to an inactivated SARS-CoV-2 vaccine in a pediatric population and explains SARS-CoV-2 serological antibody positivity in unvaccinated children.

## Data availability statement

The raw data supporting the conclusions of this article will be made available by the authors, without undue reservation.

## Ethics statement

This study has been approved by the Ethics Committee of Capital Institute of Pediatrics. It was a retrospective study, and all the participants’ data were anonymously reported in this study. Based on the guidelines of the Ethics Committee of the Capital Institute of Pediatrics, informed consent was not sought from participants.

## Author contributions

JL and LM conceived and designed the study. JL was the first author and LM was the corresponding author of the manuscript. JL and MG performed the statistical analysis and prepared the figures and table. JL wrote the first draft and LM critically revised the manuscript. SD, QS, WL, YW and WX were responsible for data collection and laboratory analyses. All authors contributed to the article and approved the submitted version.

## Conflict of interest

The authors declare that the research was conducted in the absence of any commercial or financial relationships that could be construed as a potential conflict of interest.

## Publisher’s note

All claims expressed in this article are solely those of the authors and do not necessarily represent those of their affiliated organizations, or those of the publisher, the editors and the reviewers. Any product that may be evaluated in this article, or claim that may be made by its manufacturer, is not guaranteed or endorsed by the publisher.
